# Effects of Acute Cocoa Supplementation on Postprandial Apolipoproteins, Lipoprotein Subclasses, and Inflammatory Biomarkers in Adults with Type 2 Diabetes after a High-Fat Meal

**DOI:** 10.3390/nu12071902

**Published:** 2020-06-27

**Authors:** Dustin W. Davis, Rickelle Tallent, James W. Navalta, Anthony Salazar, Timothy J. Lyons, Arpita Basu

**Affiliations:** 1 Department of Kinesiology and Nutrition Sciences, School of Integrated Health Sciences, University of Nevada, Las Vegas, NV 89154, USA; dustin.davis@unlv.edu (D.W.D.); rickelle.tallent@unlv.edu (R.T.); james.navalta@unlv.edu (J.W.N.); salaza10@unlv.nevada.edu (A.S.); 2 Division of Endocrinology, Medical University of South Carolina, Charleston, SC 29245, USA; lyonstj@musc.edu

**Keywords:** phytochemicals, functional food, dietary polyphenols, flavonoids, catechins, dyslipidemia, nutraceuticals, inflammation, chronic disease

## Abstract

Dyslipidemia and inflammation exacerbate postprandial metabolic stress in people with diabetes. Acute dietary supplementation with polyphenols shows promise in improving postprandial metabolic stress in type 2 diabetes (T2D). Cocoa is a rich source of dietary polyphenols with demonstrated cardioprotective effects in adults without diabetes. To date, the acute effects of cocoa on postprandial lipids and inflammation have received little attention in the presence of T2D. This report expands on our earlier observation that polyphenol-rich cocoa, given as a beverage with a fast-food-style, high-fat breakfast, increased postprandial high-density lipoprotein-cholesterol (HDL-C) in adults with T2D. We now test whether polyphenol-rich cocoa modulated postprandial apolipoproteins (Apo-A1, B), non-esterified fatty acids, nuclear magnetic resonance (NMR)-derived lipoprotein subclass profiles, and select biomarkers of inflammation following the same dietary challenge. We found that cocoa decreased NMR-derived concentrations of total very low-density lipoprotein and chylomicron particles and increased the concentration of total HDL particles over the 6-hour postprandial phase. Serum interleukin-18 was decreased by cocoa vs. placebo. Thus, polyphenol-rich cocoa may alleviate postprandial dyslipidemia and inflammation following a high-fat dietary challenge in adults with T2D. The study was registered at clinicaltrials.gov as NCT01886989.

## 1. Introduction

In the United States (U.S.), 88 million adults (34.5%) have prediabetes, and another 34 million (13.0%) have type 2 diabetes (T2D) [1,2]. T2D is the seventh leading cause of mortality, contributing to over a quarter-million U.S. deaths in 2017 alone [1]. The burgeoning prevalence also causes considerable financial strain. Indirect and direct expenses for treating the disease surpassed $300 million in 2017, an increase of $70 million from 2012 [1]. The health and financial burdens are projected to grow over the next four decades alongside the number of older adults, who are disproportionately affected by T2D. The population of U.S. adults aged ≥ 65 years (T2D prevalence of 26.8% [1]) is expected to grow from 50 million (16%) to 100 million (23%) by 2060 [3]. Despite efforts made in prevention, millions will still develop T2D and need treatment.

Key objectives of T2D management are the mitigation of hyperglycemia and hyperlipidemia, especially during the postprandial (fed) state. Exaggerated glycemic and lipemic excursions after food consumption damage the vascular endothelium, impairing vasodilation and causing leukocyte activation, inflammation, and intimal thickening that lead to atherosclerotic cardiovascular disease (CVD) [4,5,6,7,8,9,10,11,12]. Clinical trials have shown that elevations of fatty acids, triglycerides, and inflammatory cytokines are exacerbated by a high-fat (HF) meal [13,14] and modulated by the type of protein consumed with the meal [14]. Elsewhere it has been reported that fast-food-style meals dense in dietary energy, fat, and refined carbohydrates exacerbate postprandial triglycerides, inflammatory cytokines, and oxidative stress while decreasing cardioprotective high-density lipoprotein cholesterol (HDL-C) [15,16,17].

Knowing that meal composition directly influences the postprandial metabolic profile, researchers have explored whether acutely supplementing meals with dietary antioxidants and bioactive compounds attenuates postprandial metabolic stress. Clinical trials in patients with T2D have shown that the antioxidant vitamins C [18,19] and E [18] protected against postprandial endothelial dysfunction and oxidative stress after a HF dietary challenge. Additionally, a review concluded that fruit-derived dietary polyphenols ( from red and white wines, apples, grapes, and berries) increase plasma antioxidant capacity, possibly protecting against the pro-inflammatory, pro-oxidant state that follows a typical Western meal [20].

Cocoa, derived from cocoa beans, is a rich source of dietary polyphenols, especially epicatechin [21]. Though commercial processing of raw beans, such as Dutch processing, reduces the total polyphenol content of commercially available cocoa products, cocoa powder formulated for research, and powders that involve less processing of the natural powder, are considered a significant source of polyphenols [21,22] and are associated with cardiovascular health benefits [22,23]. A meta-analysis of studies of short-term cocoa supplementation concluded that, after two weeks, cocoa decreased low-density lipoprotein cholesterol (LDL-C) and insulin resistance, increased HDL-C, and improved endothelial function [24]. Clinical trials that employed acute supplementation of meals with cocoa have reported varied findings. One found that, following a HF meal in healthy adults, cocoa preserved endothelial function but did not affect postprandial triglycerides or fatty acids [25]. Another reported that ingestion of (-)-epicatechin (the principal polyphenol in cocoa) with an oral fat tolerance test lowered the respiratory quotient and decreased postprandial triglyceridemia in adults with normal weight and overweight, suggesting improved fat oxidation [26]. Studies in people with T2D are sparse, but we previously conducted and reported an acute clinical trial in adults with obesity and T2D who consumed a polyphenol-rich cocoa vs. placebo beverage with a fast-food-style HF breakfast: cocoa increased postprandial HDL-C but did not affect LDL-C or triglycerides [27].

Lowering LDL-C and increasing HDL-C are established goals in the prevention and treatment of T2D, CVD, and cardiac events [28]. However, approximately half of all patients hospitalized for CVD have normal LDL-C [29] and HDL-C concentrations [30]. The paradoxical co-existence of cardiometabolic disease and normal LDL-C and HDL-C levels may be due in part to variability in the size and function of LDL [31,32] and HDL particles [30]. The molar concentrations of these particles, ‘LDL-P’ and ‘HDL-P’ are not determined in a conventional lipid panel. Whereas LDL contain apolipoprotein-B (Apo-B) and are considered atherogenic, HDL contain apolipoprotein-A1 (Apo-A1) and are considered cardioprotective [33,34]. Consequently, assessing the overall concentrations of Apo-B- and Apo-A1-containing lipoproteins and the levels of different lipoprotein subclasses may provide useful added data compared with “conventional lipid profiles” when assessing CVD risk. This assertion is supported by the finding that the Apo-B:Apo-A1 ratio predicts CVD better than conventional lipid measurements [33,34,35,36]. In addition to lipoprotein characteristics, non-esterified fatty acids (NEFAs) predict CVD mortality in certain at-risk populations, such as older adults with chronic kidney disease [37]. Elevated NEFAs are widely accepted as mediators of insulin resistance, T2D, and CVD [38]. Further, in apparently healthy young adults, high postprandial NEFA levels increase arterial stiffness and the expression of adhesion molecules [38].

Considering available literature, it would seem prudent to evaluate Apo-B- and Apo-A1-containing lipoproteins, their various subclasses, NEFAs, and markers of inflammation in assessing the efficacy of cocoa on cardiovascular risk following a HF meal. To our knowledge, there are no data to address this question. Thus, using blood samples from our previously published clinical trial [27], we determined if polyphenol-rich cocoa would promote a healthier lipid profile and attenuate vascular inflammation after a fast-food-style HF meal in adults with obesity and T2D. We hypothesized that cocoa would decrease circulating Apo-B-containing lipoprotein subclasses, NEFAs, inflammatory cytokines (interleukin-6 [IL-6], interleukin-1β [IL-1β], and interleukin-18 [IL-18]), and total nitrite. We also hypothesized that cocoa would increase the concentration of Apo-A1-containing lipoproteins.

## 2. Materials and Methods

The Institutional Review Board (IRB) of the University of Oklahoma Health Sciences Center (OUHSC) and of the Oklahoma State University approved this double-blind, randomized, and controlled crossover clinical trial (IRB number: 2179). All participants signed a written informed consent form and were treated in accordance with the Declaration of Helsinki. Study recruitment and data collection took place at the Harold Hamm Diabetes Center at OUHSC. The study was registered at clinicaltrials.gov as NCT01886989. The participants and methods of this clinical trial were stated in detail elsewhere [27] but are described briefly below.

Eighteen adults ≥ 21 years of age completed all study procedures. All had a history of five or more years of abdominal adiposity and T2D. None were treated with insulin. People with known CVD risk factors (e.g., hypertension or dyslipidemia) and/or taking oral medications for diabetes or other risks were included. Exclusion criteria were cancer; CVD event; abnormal liver, renal, or thyroid function; currently attempting weight loss; regularly ingesting antioxidants or fish oil; smoking or alcohol consumption; and pregnancy or lactation. Participants completed two HF meal challenges separated by one week. They were randomized to receive a polyphenol-rich cocoa or placebo beverage, and pre- and postprandial samples were collected at fasting and up to six hours (h). Before arrival on both testing days, participants were instructed to abstain from alcohol and caffeine for 24 h and from polyphenol-rich foods or dietary supplements for 48 h. Aside from these restrictions, we encouraged participants to adhere to their normal diet and medication regimen and level of physical activity. Dietary habits were documented using a three-day food record at baseline.

During each test day, blood samples were collected at baseline (after fasting for 10–12 h) and at 0.5 h, 1 h, 2 h, 4 h, and 6 h after a fast-food-style HF meal comprised of scrambled eggs, hash brown potatoes, a sausage patty, buttermilk biscuits, and butter. In total, the meal contained 766 kilocalories (kcal), 59% of energy as fats (50 g [g] total fats, 25 g saturated fats, 12 g monounsaturated fats, and 13 g polyunsaturated fats), 26% of energy as carbohydrates (50 g) and 16% as protein (30 g), 465 milligrams (mg) of cholesterol, and 2.4 g of dietary fiber. The Hershey Company (Hershey, PA, USA) provided the cocoa and placebo powders, which were reconstituted in water for administration as a beverage and were matched for kcal, fat, and macronutrient distribution but differed by fiber, caffeine, total polyphenols, total flavanols, proanthocyanins, epicatechins, and theobromine as previously described [27]. The 20 g cocoa powder provided a total of 960 mg polyphenols, 480 mg flavanols, 201 mg proanthocyanidins, and 40 mg epicatechin, whereas the 12 g placebo powder had negligible amounts of these bioactive compounds. Plasma samples were sent for determination of nuclear magnetic resonance (NMR)-derived Lipoprotein Subclass Profiles (NMR-LSP) in first-thaw specimens (250 microliters) using a 400-MHz proton NMR analyzer at LipoScience Inc. (Raleigh, NC, USA) as described previously [39]. Serum Apo-B, Apo-A1, and NEFAs were measured using standard clinical chemistry techniques on a COBAS Integra 400 plus analyzer according to the manufacturer’s instructions (Roche Diagnostics, Rotkreuz, Switzerland). Serum IL-6, IL-1β, and IL-18 were determined using ELISA kits based on the manufacturer’s protocol (R&D Systems, Minneapolis, MN, USA) with inter-assay coefficients of variation (CV) of 5.5%, 7.2%, and 7.5%, respectively. Serum nitrite was measured using the Griess Reagent System (Promega Corporation, Madison, WI, USA) with a mean inter-assay CV of 4.1%.

The biomarker concentrations at fasting and at each postprandial time point are presented as mean ± standard error of the mean (SEM) unless stated otherwise. We used a mixed model ANCOVA while adjusting for fasting values as covariates to examine changes in variables over 6 h of the postprandial period, differences between intervention phases (cocoa vs. placebo), and whether overall changes in time differed between cocoa and placebo phases. Data analyses were conducted with the use of IBM SPSS Statistics version 20.0 (IBM Corp., Armonk, NY, USA). Results corresponding to *p* < 0.05 are described as significant for the purposes of discussion.

## 3. Results

Eighteen adults completed the cocoa and placebo phases of the randomized crossover trial. The baseline characteristics of participants have been previously published [27]. Briefly, all had obesity (body mass index [BMI] > 30.0 kg/m^2^) with hyperglycemia in the T2D range, mostly managed by oral hypoglycemic agents (83%), and few (23%) were on statins/fibrates to lower lipids. Among the 18 participants, there were four males and 14 females with mean (± SE) age of 56 ± 3 years. None reported any changes in dietary habits or lifestyle changes throughout the short postprandial trial.

As shown in Table 1, postprandial serum Apo-B did not significantly change over time and did not differ between the polyphenol-rich cocoa and placebo interventions. Postprandial serum Apo-A1 tended to increase over six hours in the postprandial phase (*p* = 0.06), but this change did not significantly differ between the cocoa and placebo interventions. No significant changes were noted in the Apo-B:Apo-A1 ratio. Serum NEFAs revealed a significant change over 6 h (*p* < 0.001) and tended to be higher in the cocoa vs. placebo phase (*p* = 0.07).

As illustrated in Table 2, the concentration of NMR-derived total very low-density lipoprotein (VLDL) and chylomicron particles significantly increased across the 6-h postprandial phase (*p* = 0.02) but remained lower with the polyphenol-rich cocoa intervention than placebo (*p* = 0.03). The cocoa phase also tended to decrease small VLDL particles more than placebo (*p* = 0.07). No overall significant effects were noted for large and medium VLDL (*p* > 0.05). Similarly, cocoa or placebo intervention in the presence of a fast-food-style HF meal did not affect concentrations of any of the LDL subclasses (*p* > 0.05). Cocoa significantly increased total HDL-P over the 6-h postprandial phase compared to the placebo (*p* = 0.04). Large HDL, medium HDL, and small HDL-P did not significantly differ across time or between interventions, nor did the size of VLDL, LDL, or HDL (*p* > 0.05).

Finally, Table 3 shows that IL-6 tended to change during the 6-h postprandial period (*p* = 0.06) but did not differ between the polyphenol-rich cocoa and the placebo phases (*p* > 0.05). Neither IL-1β nor nitrite were significantly affected by either intervention, but nitrite tended to increase over time (*p* = 0.08). The cocoa powder significantly affected IL-18, which increased after placebo but decreased after cocoa (*p* < 0.001).

## 4. Discussion

Our clinical investigation was conducted considering accumulating evidence that dietary cocoa confers health benefits [22,23]. Cocoa is abundant in various dietary polyphenols and is a richer source of flavonoids than other commonly consumed beverages such as green tea and red wine [40]. Epicatechin is the predominant flavonoid in cocoa, but others are also present, including flavan-3-ols, anthocyanins, and flavones that are associated with improvements in lipemia [23], an important target in slowing the progression of atherosclerotic CVD in T2D [28]. A meta-analysis showed that short-term polyphenol-rich cocoa consumption (duration of intervention ranging from 14 to 126 days) decreased LDL-C by approximately 2.98 mg/dL and increased HDL-C by 1.78 mg/dL [24].

We examined the acute effects of polyphenol-rich cocoa on postprandial lipemia: the supplement was consumed with a single fast-food-style HF meal. To date, clinical trials on this topic are scarce. One showed that cocoa attenuated endothelial dysfunction but did not improve postprandial triglycerides or fatty acids in healthy adults with normal weight who consumed a liquid HF meal (3 mL whipping cream [= 1 g fat] per kg body weight) [25]. Another showed that epicatechin taken with a liquid oral tolerance test meal (246 kcal, 6 g fat, 39 g carbohydrates, 9 g protein) mitigated postprandial triglycerides and fat oxidation in adults with normal weight and overweight [26]. We previously reported conventional lipid data from the present clinical trial showing that polyphenol-rich cocoa increased postprandial HDL-C [27]. In the current report, we report further details not previously published on postprandial apolipoproteins, NEFAs, and NMR-derived LSP after acute cocoa supplementation in adults with T2D. In addition, we report select inflammatory biomarkers known to be modulated by dietary challenges. Our data showed that cocoa significantly suppressed the postprandial rise in total VLDL and chylomicron particle concentrations. Further, cocoa was associated with a reduction in postprandial IL-18, contrasting with an increase following placebo, and it attenuated the postprandial decline in total HDL-P. Cocoa had no effect on postprandial Apo-A1, Apo-B, or the Apo-B:Apo-A1 ratio and was associated with a non-significant trend towards higher NEFA levels compared to placebo.

The reduction in total VLDL and chylomicron particles is clinically meaningful. Based on an NMR analysis of samples in people with newly-diagnosed T2D, Amor et al. reported molar VLDL particle concentration to be linearly associated with the number of common carotid plaques (extended into lumen by ≥ 50% of surrounding intima-media thickness value or 1.5 millimeters) [41]. Separately, Mora et al. followed 27,763 women for 11 years and found that the NMR-derived VLDL particle concentration was an independent predictor of cardiovascular events [42]. Finally, in a metabolite profiling study with 15 years of follow-up, each standard deviation increase in log-transformed VLDL particle concentration increased the hazard ratio for a cardiac event by 1.22 after controlling for age, sex, blood pressure, smoking, and diabetes [43].

Polyphenol-rich cocoa may have attenuated the postprandial rise in VLDL and chylomicron particles by interfering with their synthesis, which is typically increased in the presence of insulin resistance [44]. Also, catechins have been shown to inhibit intestinal lipid absorption in animal and epidemiological studies [45]. In an animal study employing a liquid dietary challenge, rats supplemented with 0.1 or 0.5 g epigallocatechin gallate per kg body mass absorbed only 73.7 and 62.7% of intestinal cholesterol, respectively, compared to the control rats that absorbed 79.3% [46]. Other studies in animal models of obesity and diabetes demonstrate the effects of cocoa extract in reducing serum and hepatic triglycerides. In a diet-induced obesity model of Wistar rats, ten weeks of cocoa extract supplementation (14 and 140 mg per kg body mass per day) revealed a significant decrease in serum and hepatic triglyceride content [47]. In another study of rats with obesity and diabetes, cocoa extract supplementation (600 mg per kg body mass) for four weeks decreased plasma triglycerides compared to the non-supplemented group [48]. Cocoa flavonols, especially the procyanidins, have been shown to be potent inhibitors of lipid digestive enzymes [49], thereby decreasing the intestinal absorption of lipids, such as plasma triglycerides, and molar concentrations of VLDL particles as revealed by NMR-LSP in our clinical study. Further mechanistic studies are needed to determine the effects of cocoa on postprandial lipids. Nonetheless, our clinical trial shows that polyphenol-rich cocoa with a HF meal is a promising acute dietary intervention for postprandial VLDL and chylomicron particle excursions in people with obesity and T2D.

Another key finding of this study is that polyphenol-rich cocoa taken with a fast-food-style HF meal mitigated the postprandial reduction in molar HDL-P concentrations. This observation aligns with our previous finding that cocoa increased the conventional lipid measurement of postprandial HDL-C by 1.5 ± 0.8 mg/dL compared to the placebo [27]. While the two measures are associated, HDL-C and molar HDL-P concentration reflect distinct characteristics of the lipoprotein, and there is evidence that particle concentration may be the superior measure of coronary heart disease (CHD, a form of CVD) risk [50]. In the Veterans Affairs High-Density Lipoprotein Intervention Trial (VA-HIT), men with a new CHD event were followed for five years. Each one standard deviation increase in HDL-P concentration decreased CHD risk by 29%. In contrast, neither HDL-C nor Apo-A1 were significant predictors [51]. Separately, the Multiple Risk Factor Intervention Trial (MRFIT) tracked men with metabolic syndrome for 18 years. While baseline HDL-C did not predict death from CHD, risk was approximately 50% lower among those with baseline HDL-P concentrations in the top quartile compared to the bottom quartile [52]. The European Prospective Investigation into Cancer and Nutrition (EPIC)-Norfolk reported similar findings in apparently healthy men and women: those in the top quartile for HDL-P concentration had an approximately 50% reduced risk of coronary artery disease compared to those in the bottom quartile after controlling for the concentrations of triglycerides and Apo-B [53]. Considering these clinical trials and the evidence we present here, we contend that polyphenol-rich cocoa conferred cardio-protection by sustaining HDL-P concentrations and HDL-C concentrations for 6 h after a fast-food-style HF meal.

Protection against atherosclerotic CVD may also be achieved by reducing inflammation, an essential process in the progression of atherosclerosis and CHD [54]. Postprandial hyperglycemia and hyperlipidemia exacerbate postprandial systemic inflammation and vasoconstriction. The degree of response can be assessed by measuring select inflammatory biomarkers and nitrite that are sensitive to dietary challenges [55]. In randomized controlled clinical trials, acute interventions with dietary polyphenols have shown promise in mitigating postprandial inflammation. For example, cranberries and raspberries dampened postprandial elevations of serum IL-6 and IL-18, respectively, in adults with obesity and T2D after a HF breakfast [56,57]. Further, Oh et al. recently showed that a spice blend supplement (6 g) taken with a HF, high-carbohydrate meal reduced IL-1β, IL-8, and tumor necrosis factor-α secretion by liposaccharide-stimulated (LPS) peripheral blood mononuclear cells of men who had overweight or obesity [58]. In the present study, polyphenol-rich cocoa did not affect serum IL-6, IL-1β, or nitrite but lowered IL-18 compared to the placebo. Our observation of this modest change in inflammatory biomarkers, confined to serum IL-18, may be explained by the background diet of the participants, bioactive components of cocoa compared to other foods (e.g., dietary berries), the type of dietary challenge, and our selection of the serum cytokines to assess postprandial changes. A 2016 review reported that supplementation of cocoa-containing foods or beverages improved postprandial inflammatory biomarkers in some instances (e.g., intercellular adhesion molecule-1 [ICAM-1] and endothelial E-selectin in healthy adults) [59]. However, the findings were not consistent across interventions and samples. Separately, in a clinical trial where adults with obesity and risk for insulin resistance consumed a cocoa beverage (vs. a low-flavanol control beverage) for five consecutive days, ICAM-1, IL-6, and C-reactive protein were reduced after a 75-g glucose load [60]. Given that dietary cocoa has shown potential anti-inflammatory effects when provided acutely as a single bolus or over a short timeframe, additional clinical investigations are merited.

Our randomized, controlled, and crossover clinical trial has several noteworthy strengths. Importantly, we are first to describe how apolipoproteins, NEFAs, NMR-derived LSP, and inflammatory cytokines were affected by consuming a dietary achievable dose of polyphenol-rich cocoa (20 g) with a fast-food-style HF meal. Additionally, we provided the cocoa powder reconstituted in plain water that had no other added ingredients, which suggests that our findings are attributable to cocoa alone. Another strength was our inclusion of participants with obesity and T2D who had CVD risks and took oral hypoglycemic agents, thus enhancing the generalizability of our findings to the broader population with an impaired cardiometabolic profile. There are also some limitations of our study. First, our small sample size may have affected the detection of significant differences in this exploratory analysis. Second, the lack of a control group without diabetes precludes us from extrapolating our findings to any population other than adults with obesity and T2D. Third, because we provided cocoa with only one type of meal challenge (Western breakfast foods high in total and saturated fat), our findings do not address the effects of cocoa when consumed with meals containing different foods or a smaller fat content. Also, cocoa powder contains a large variety of nutritional compounds in addition to polyphenols, such as xanthines, minerals, fiber, and fatty acids which must be examined in future metabolic trials. Fourth, based on the acute nature of our intervention, we could not elucidate the hepatic and adipose responses which may be revealed by assessment of postprandial absorptive phase beyond six hours. Lastly, our participants did not have overt CVD and were managing glycemia through diet, physical activity, and oral drugs. Nonetheless, our study has practical relevance because it tested the effects of a fast-food-style HF breakfast commonly consumed by U.S. adults. Therefore, our findings may be inapplicable to adults with uncontrolled T2D and/or major micro- and macrovascular complications.

## 5. Conclusions

**Figure 1:**
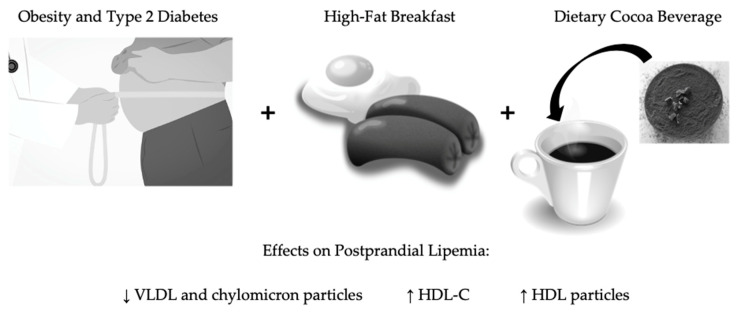
Effects of acute polyphenol-rich cocoa supplementation on postprandial lipemia after a fast-food-style, high-fat Western breakfast in adults with obesity and type 2 diabetes. VLDL: very low-density lipoprotein; HDL-C: high density lipoprotein cholesterol; HDL-P: high-density lipoprotein particles.

Postprandial dyslipidemia and inflammation are implicated in atherosclerosis and CVD. Conventional lipid measurements after a dietary challenge do not reveal the full extent of quantitative changes in blood lipids. Analyses of apolipoproteins, NEFAs, and NMR-derived LSP may better indicate cardiovascular risk in susceptible populations. Consequently, NMR is a valuable tool for detecting changes in lipids, beyond those revealed by a conventional lipid analysis, following dietary interventions targeting postprandial lipids. In the present study, we showed that a polyphenol-rich cocoa beverage with a fast-food-style HF breakfast decreased postprandial VLDL and chylomicron particles and increased HDL-P concentrations over 6 h in adults with obesity and T2D. We additionally showed that cocoa reduced IL-18, thus potentially alleviating postprandial inflammation (Figure 1). Future studies would benefit from recruiting larger numbers of participants and evaluating lipid outcomes using NMR-derived LSP in addition to a conventional lipid panel. New considerations should include the efficacy of different cocoa doses; the foods and fat content used in dietary challenges; and testing cocoa interventions in samples with different demographic and metabolic characteristics, such as advanced T2D and cardiovascular complications.

## Figures and Tables

**Table 1:** Postprandial serum levels of apolipoproteins and non-esterified fatty acids in adults with type 2 diabetes following a high-fat meal challenge with polyphenol-rich cocoa or placebo beverage.

Variable	Intervention	Fasting	1 h	2 h	4 h	6 h	Time (*p*)	Intervention (*p*)	Time * Intervention (*p*)
Apo-B (mg/dL)	Cocoa	96 ± 5	94 ± 5	97 ± 5	94 ± 5	100 ± 5	0.70	0.57	0.58
	Placebo	96 ± 4	93 ± 5	95 ± 4	97 ± 5	98 ± 4			
Apo-A1 (mg/dL)	Cocoa	158 ± 8	153 ± 7	155 ± 6	167 ± 8	166 ± 8	0.06	0.97	0.26
	Placebo	155 ± 6	167 ± 8	153 ± 8	162 ± 9	164 ± 8			
Apo-B:Apo-A1 Ratio	Cocoa	0.63 ± 0.04	0.63 ± 0.04	0.64 ± 0.04	0.59 ± 0.04	0.63 ± 0.05	0.48	0.92	0.51
	Placebo	0.63 ± 0.03	0.57 ± 0.04	0.66 ± 0.04	0.63 ± 0.05	0.62 ± 0.04			
NEFAs (mg/dL)	Cocoa	0.62 ± 0.04	0.49 ± 0.04	0.44 ± 0.04	0.51 ± 0.05	0.65 ± 0.05	**<0.001** ^1^	0.70	0.07
	Placebo	0.64 ± 0.05	0.42 ± 0.05	0.36 ± 0.03	0.45 ± 0.03	0.71 ± 0.06			

Data presented as mean ± SEM. *indicates the time by intervention interaction effect; Apo-B: apolipoprotein-B; mg: milligrams; dL: deciliter; *p*: probability value; Apo-A1: apolipoprotein-A1; Apo-B:Apo-A1 ratio: ratio of Apo-B to Apo-A1; NEFA: non-esterified fatty acids; ^1^ significant effect of time with *p* < 0.05; *p*-values derived from linear mixed model ANCOVA adjusted for fasting values as covariates.

**Table 2:** Postprandial plasma levels of NMR-derived lipoprotein subclasses in adults with type 2 diabetes following a high-fat meal challenge with polyphenol-rich cocoa or placebo beverage.

Variable	Intervention	Fasting	1 h	2 h	4 h	6 h	Time (*p*)	Intervention (*p*)	Time * Intervention (*p*)
Total VLDL and Chylomicron Particles (nmol/L)	Cocoa	39.1 ± 17.9	38.2 ± 15.8	46.1± 12.8	47.9 ± 19.2	45.7 ± 19.6	0.02 ^1^	0.08	0.03 ^2^
	Placebo	43.1 ± 18.2	40.1 ± 19.8	47.9 ± 18.8	62.2 ± 21.5	58.6 ± 15.4			
Large VLDL and Chylomicron Particles (nmol/L)	Cocoa	5.7 ± 3.8	7.1 ± 3.7	8.9 ± 4.4	7.7 ± 5.1	7.2 ± 5.8	0.31	0.23	0.38
	Placebo	6.6 ± 5.0	6.2 ± 4.4	5.9 ± 3.5	8.2 ± 4.4	8.1 ± 5.3			
Medium VLDL Particles (nmol/L)	Cocoa	12.6 ± 10.5	14.5 ± 10.6	19.1 ± 13.6	18.0 ± 14.2	14.8 ± 9.7	0.21	0.19	0.37
	Placebo	16.7 ± 10.5	15.8 ± 14.5	11.1 ± 10.6	28.5 ± 14.1	20.9 ± 14.7			
Small VLDL Particles (nmol/L)	Cocoa	20.9 ± 9.9	16.5 ± 11.5	18.1 ± 11.6	22.2 ± 17.0	23.7 ± 14.5	0.18	0.08	0.07
	Placebo	19.9 ± 13.2	18.0 ± 12.0	31.0 ± 13.5	24.2 ± 15.2	24.7 ± 12.4			
Total LDL Particles (nmol/L)	Cocoa	1120 ± 325	1136 ± 320	1221 ± 202	1018 ± 290	1092 ± 306	0.41	0.32	0.30
	Placebo	1083 ± 195	997 ± 245	1100 ± 278	1056 ± 226	1049 ± 267			
IDL Particles (nmol/L)	Cocoa	333 ± 173	280 ± 172	167 ± 144	336 ± 179	264 ± 186	0.48	0.26	0.19
	Placebo	277 ± 103	283 ± 206	383 ± 141	211 ± 168	328 ± 201			
Large LDL Particles (nmol/L)	Cocoa	209 ± 152	349 ± 237	410 ± 151	182 ± 157	299 ± 191	0.41	0.29	0.15
	Placebo	317 ± 170	250 ± 199	263 ± 145	195 ± 169	184 ± 168			
Total Small LDL Particles (nmol/L)	Cocoa	686 ± 251	600 ± 256	726 ± 198	596 ± 289	582 ± 241	0.34	0.23	0.38
	Placebo	594 ± 268	542 ± 178	685 ± 256	649 ± 279	537 ± 249			
Total HDL Particles (µmol/L)	Cocoa	29.9 ± 5.0	26.1 ± 4.1	27.3 ± 4.3	27.2 ± 6.6	26.0 ± 5.8	0.15	0.08	0.04 ^2^
	Placebo	28.0 ± 3.1	25.3 ± 7.5	24.6 ± 6.5	24.3 ± 3.8	23.1 ± 5.8			
Large HDL Particles (µmol/L)	Cocoa	4.3 ± 1.9	3.4 ± 2.0	4.3 ± 1.8	3.1 ± 1.9	4.7 ± 2.3	0.21	0.26	0.33
	Placebo	4.0 ± 2.1	3.3 ± 2.9	4.0 ± 2.4	5.0 ± 2.8	4.0 ± 2.5			
Medium HDL Particles (µmol/L)	Cocoa	5.6 ± 4.8	4.9 ± 3.8	6.0 ± 3.8	6.4 ± 4.5	6.4 ± 4.4	0.25	0.31	0.45
	Placebo	5.5 ± 3.4	3.8 ± 2.2	5.5 ± 3.1	6.3 ± 4.2	4.2 ± 3.7			
Small HDL Particles (µmol/L)	Cocoa	19.2 ± 5.6	17.7 ± 4.3	17.1 ± 4.1	15.7 ± 4.8	15.0 ± 4.8	0.36	0.28	0.29
	Placebo	17.5 ± 1.4	18.2 ± 4.8	15.1 ± 4.3	16.0 ± 4.1	14.8 ± 3.4			
VLDL Size (nm)	Cocoa	55.7 ± 7.2	59.7 ± 8.2	59.6 ± 8.3	57.2 ± 11.3	56.9 ± 12.9	0.26	0.22	0.43
	Placebo	56.7 ± 7.5	55.1 ± 7.9	52.2 ± 5.1	57.1 ± 7.4	55.6 ± 9.0			
LDL Size (nm)	Cocoa	20.0 ± 0.6	20.5 ± 0.8	20.6 ± 0.7	20.0 ± 0.6	20.6 ± 0.7	0.32	0.44	0.53
	Placebo	20.6 ± 0.9	20.4 ± 0.8	20.0 ± 0.5	20.3 ± 0.5	20.6 ± 0.8			
HDL Size (nm)	Cocoa	9.2 ± 0.5	9.0 ± 0.6	9.1 ± 0.4	9.0 ± 0.4	9.2 ± 0.6	0.26	0.42	0.70
	Placebo	9.2 ± 0.5	9.0 ± 0.7	9.2 ± 0.5	9.2 ± 0.5	9.3 ± 0.5			

Data presented as mean ± SEM. *indicates the time by intervention interaction effect; NMR: nuclear magnetic resonance; VLDL: very low density lipoprotein; nmol: nanomoles; L: liter; *p*: probability value; LDL: low density lipoprotein; IDL: intermediate density lipoprotein; HDL: high density lipoprotein; µmol: micromoles; nm: nanometers; ^1^ significant effect of time with *p* < 0.05; ^2^ significant time * intervention effect with *p* < 0.05; *p*-values derived from linear mixed model ANCOVA adjusted for fasting values as covariates.

**Table 3:** Postprandial serum levels of inflammatory cytokines and nitrite in adults with type 2 diabetes following a high-fat meal challenge with a polyphenol-rich cocoa or placebo beverage.

Variable	Intervention	Fasting	1 h	2 h	4 h	6 h	Time (*p*)	Intervention (*p*)	Time * Intervention (*p*)
IL-6 (pg/mL)	Cocoa	3.2 ± 0.5	5.1 ± 0.7	6.8 ± 2.4	3.1 ± 0.6	4.5 ± 1.3	0.06	0.39	0.51
	Placebo	6.3 ± 2.1	4.4 ± 1.2	7.6 ± 2.5	5.4 ± 1.8	5.0 ± 2.6			
IL-1β (pg/mL)	Cocoa	3.0 ± 0.4	5.5 ± 2.0	3.6 ± 0.4	6.3 ± 1.1	4.0 ± 0.4	0.55	0.15	0.77
	Placebo	3.5 ± 1.1	2.4 ± 0.4	3.1 ± 1.3	3.0 ± 1.0	4.2 ± 0.6			
IL-18 (pg/mL)	Cocoa	300 ± 12	279 ± 11	286 ± 12	267 ± 9	250 ± 9	0.45	0.001 ^1^	<0.001 ^2^
	Placebo	303 ± 13	302 ± 11	290 ± 12	325 ± 13	339 ± 15			
Nitrite (µM)	Cocoa	6.2±1.5	11.1±2.6	13.1±1.6	13.7±2.3	12.2±2.3	0.08	0.12	0.43
	Placebo	8.3±4.5	10.6±4.0	14.2±3.5	17.7±4.0	13.8±3.0			

Data presented as mean ± SEM. * indicates the time by intervention interaction effect; IL-6: interleukin-6; pg: picograms; mL: milliliter; *p*: probability value; IL-1β: interleukin-1β; IL-18: interleukin-18; ^1^ significant effect of intervention with *p* < 0.05; ^2^ significant time * intervention effect with *p* < 0.05; *p*-values derived from linear mixed model ANCOVA adjusted for fasting values as covariates.4.
